# Eating quickly is associated with a low aspartate aminotransferase to alanine aminotransferase ratio in middle-aged adults: a large-scale cross-sectional survey in Japan

**DOI:** 10.1186/s13690-020-00482-3

**Published:** 2020-10-16

**Authors:** Eri Ozaki, Hirotaka Ochiai, Takako Shirasawa, Takahiko Yoshimoto, Satsue Nagahama, Jun Muramatsu, Takahiko Chono, Takayoshi Ito, Haruhiro Inoue, Akatsuki Kokaze

**Affiliations:** 1 Department of Hygiene, Public Health and Preventive Medicine, Showa University School of Medicine, 1-5-8 Hatanodai, Shinagawa-ku, Tokyo, 142-8555 Japan; 2 Digestive Diseases Center, Showa University Koto Toyosu Hospital, 5-1-38 Toyosu, Koto-ku, Tokyo, 135-8577 Japan; 3 All Japan Labor Welfare Foundation, 6-16-11 Hatanodai, Shinagawa-ku, Tokyo, 142-0064 Japan

**Keywords:** Eating quickly, Alanine aminotransferase, Aspartate aminotransferase to alanine aminotransferase ratio

## Abstract

**Background:**

An elevated alanine aminotransferase (ALT) and a low aspartate aminotransferase (AST) to ALT ratio (AST/ALT ratio) suggest nonalcoholic fatty liver disease and nonalcoholic steatohepatitis, increasing the risk of liver cirrhosis and hepatocellular carcinoma. In addition, eating quickly has been found to be associated with outcomes such as obesity. This study sought to investigate the relationship between eating quickly and an elevated ALT or a low AST/ALT ratio in Japanese middle-aged adults.

**Methods:**

The present study included 283,073 adults aged 40–64 years who had annual health checkups in Japan from April 2013 to March 2014. The data of serum parameters and lifestyle factors, including eating speed, were analyzed. An elevated ALT was defined as > 40 U/L, and a low AST/ALT ratio was defined as < 1. Logistic regression analysis was performed to calculate the odds ratios (ORs) and the 95% confidence intervals (CIs) for an elevated ALT and a low AST/ALT ratio.

**Results:**

Significantly increased ORs for an elevated ALT were observed in men (OR: 1.45, 95% CI: 1.41–1.49) and women (OR: 1.34, 95% CI: 1.25–1.43). Moreover, eating quickly significantly increased the ORs for a low AST/ALT ratio in men (OR: 1.53, 95% CI: 1.50–1.56) and women (OR: 1.36, 95% CI: 1.31–1.41). When the analysis was limited to those with ALT ≤40 U/L, eating quickly had significantly increased ORs for a low AST/ ALT ratio, regardless of sex.

**Conclusions:**

Eating quickly was significantly associated with an elevated ALT and a low AST/ALT ratio. In addition, eating quickly was significantly associated with a low AST/ALT ratio even for those without ALT elevation. This study suggested that modification of eating speed may contribute to reducing the risk for an elevated ALT and a low AST/ALT ratio.

## Background

Nonalcoholic steatohepatitis (NASH) is a progressive form of nonalcoholic fatty liver disease (NAFLD); it often develops into liver cirrhosis and can increase the risk for hepatocellular carcinoma [1]. Therefore, early detection of NASH is important for the prevention of liver cirrhosis and hepatocellular carcinoma. NAFLD and NASH are diagnosed based on the presence of steatohepatitis on liver biopsy [1]. An elevated alanine amino transferase (ALT) is commonly used as a surrogate marker of NAFLD [2]. In addition, an aspartate amino transferase (AST) to ALT ratio (AST/ALT ratio) of < 1 is indicative of NASH [3]. Because screening is easier with the use of blood tests than with obtaining biopsies, the use of ALT and the AST/ALT ratio may be effective for early detection of NAFLD/ NASH in a general population.

The epidemiology and demographic characteristics of NAFLD usually parallel the prevalence of obesity. Furthermore, individuals with NAFLD have a high frequency of metabolic syndrome [4]. The previous studies found that eating quickly was associated with obesity [5] and metabolic syndrome [6], because eating quickly leads to excess energy intake [7]. Thus, reducing eating speed may have a positive impact on health.

Recent studies showed that ALT was positively associated with the eating speed in Japanese men and women [8, 9]. However, these studies did not examine the association between eating quickly and ALT elevation, a surrogate marker of NAFLD. In addition, there have been no studies of the association between eating quickly and the AST/ALT ratio. If there are associations between eating quickly and an elevated ALT and a low AST/ALT ratio, then it may be possible to prevent NAFLD/NASH by modifying the eating speed.

Therefore, this study sought to examine the relationships of eating quickly with an elevated ALT and a low AST/ALT ratio using a large dataset from health checkups in Japanese men and women.

## Methods

### Subjects

**Fig. 1:**
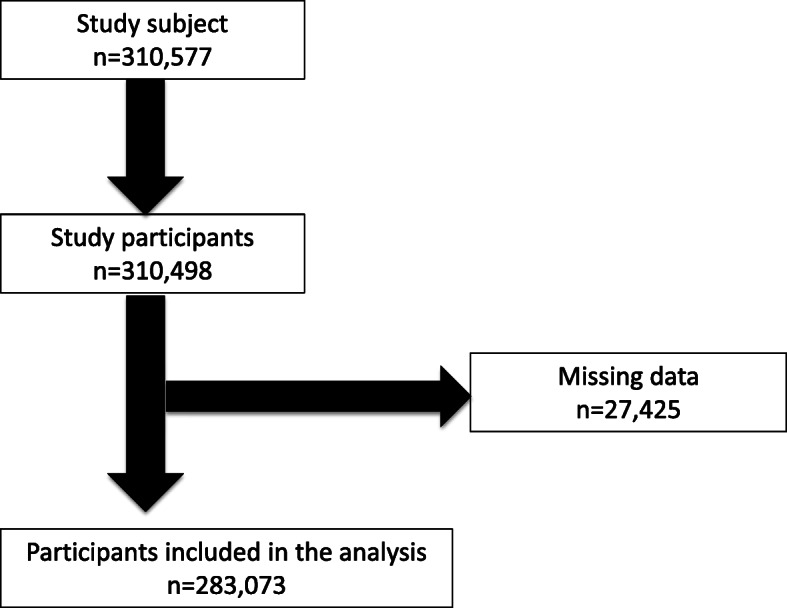
Flow diagram of study participants included in the analysis

This was a cross-sectional study that included men and women aged 40–64 years who underwent annual health checkups offered by the All Japan Labor Welfare Foundation, which is a health service center, from April 2013 to March 2014. Of 310,577 subjects, 310,498 participated in this study. A total of 27,425 participants with missing data, even partially, were excluded. Therefore, 283,073 participants (185,524 men and 97,549 women) were analyzed in this study (Fig. 1). Written informed consent was obtained from the participants. The study protocol was approved by the Medical Ethics Committee of Showa University School of Medicine (Approval No. 2717) and the Ethics Committee of the All Japan Labor Welfare Foundation (Approval No. 13-1-0011).

### Data collection

Data on biological sex (male, or female), eating speed, smoking status (current, former, or never), alcohol intake (everyday, sometimes, or none), and daily physical activity that was equivalent to walking (< 60 min, or ≥ 60 min) were obtained from a self-administered questionnaire that was recommended for a specific health examination by the Ministry of Health, Labour and Welfare. Based on the answers to the question: “Do you eat quickly compared with other people”, the information on eating speed was collected and assigned to 3 categories of quickly, normal, or slow. The height and weight of each participant were measured to the nearest 0.1 cm and 0.1 kg, respectively. The body mass index (BMI) was calculated as weight in kilograms divided by the height in square meters. The participants’ venous blood samples were collected and stored in a cooler at 4 °C for transportation to an external laboratory (SRL, Tokyo, Japan). AST, ALT, and γ-glutamyl transpeptidase (γ-GTP) levels were measured by a modified Japan Society of Clinical Chemistry reference method (AU5400, Beckman Coulter, CA, USA).

### Definitions of an elevated ALT and a low AST/ ALT

An elevated ALT was defined as > 40 U/L, according to a previous study [2]. A low AST/ALT ratio was defined as < 1, in accordance with a previous study [3].

### Statistical analysis

Data were presented as medians (25th percentile, 75th percentile) for continuous variables or as *n* (%) for categorical variables. Based on a previous study [10], eating speed was categorized into 2 groups for analysis, as follows: eating quickly (quickly) or not eating quickly (normal or slow). The Mann-Whitney U test or the chi-squared test was used to compare the characteristics between the eating quickly group and the not eating quickly group. Logistic regression analysis was used to calculate the odds ratio (OR) of eating quickly and the 95% confidence interval (CI). A crude OR was calculated in the 1st model. In the 2nd model, age, γ-GTP, smoking status, alcohol intake, and physical activity were included to control for potential confounding factors [11–14]. In the 3rd model, BMI was added to the variables included in the 2nd model (age, γ-GTP, smoking status, alcohol intake, and physical activity) for adjustment. A *P* value of < 0.05 was considered as statistically significant. All statistical analyses were performed by JMP® 15 (SAS Institute Inc., Cary, NC, USA) and Statistical Analysis System (SAS) software (version 9.4; SAS Institute Inc., Cary, NC, USA).

## Results

The characteristics of study participants according to the speed of eating are shown in Tables 1 and 2. ALT was significantly higher in the eating quickly group than in the not eating quickly group for both men (*p* <  0.01) and women (p <  0.01). On the other hand, the AST/ALT ratio was significantly lower in the eating quickly group than in the not eating quickly group for both men (*p* <  0.01) and women (p <  0.01). There were significant differences in smoking status, alcohol intake, and physical activity between the eating quickly and the not eating quickly groups (p <  0.01).

**Table 1:** Characteristics of study participants by eating quickly or not (Japan, from April 2013 to March 2014)

Eating speed		Quickly	Not quickly	z score^a^	p value^a^
Men		(*n* = 53,533)	(*n* = 131,991)		
	Age (years)	49 (44, 55)	51 (45, 57)	−39.133	< 0.001
	Height (cm)	170.2 (166.0, 174.2)	169 (164.8, 173.1)	37.668	< 0.001
	Weight (kg)	70.8 (63.8, 78.9)	66 (59.6, 73.2)	84.887	< 0.001
	BMI (kg/m2)	24.4 (22.3, 26.9)	23.1 (21.2, 25.3)	76.513	< 0.001
	AST (U/L)	23 (19, 28)	22 (19, 27)	11.314	< 0.001
	ALT (U/L)	24 (18, 35)	22 (16, 31)	37.636	< 0.001
	AST/ALT ratio	0.94 (0.74, 1.17)	1.03 (0.82, 1.27)	− 48.776	< 0.001
	γ-GTP (U/L)	36 (24, 59)	34 (23, 58)	13.173	< 0.001
Women		(*n* = 22,162)	(*n* = 75,387)		
	Age (years)	50 (45, 57)	51 (45, 57)	−4.207	< 0.001
	Height (cm)	157.6 (153.7, 161.8)	156.7 (152.9, 160.7)	20.310	< 0.001
	Weight (kg)	56.5 (50.8, 64.2)	53.2 (48.0, 59.9)	45.204	< 0.001
	BMI (kg/m2)	22.7 (20.5, 25.6)	21.6 (19.7, 24.1)	39.01	< 0.001
	AST (U/L)	20 (17, 24)	20 (17, 23)	3.953	< 0.001
	ALT (U/L)	16 (12, 22)	15 (12, 21)	14.383	< 0.001
	AST/ALT ratio	1.22 (1.00, 1.46)	1.27 (1.06, 1.50)	−18.766	< 0.001
	γ-GTP (U/L)	19 (14, 29)	19 (14, 28)	7.789	< 0.001

*BMI* body mass index, *AST* aspartate amino transferase, *ALT* alanine amino transferase

γ-GTP, γ-glutamyl transpeptidase

Data are presented as median(25th percentile,75th percentile)

^a^Mann-Whitney U test

**Table 2:** Characteristics of study participants by eating quickly or not (Japan, from April 2013 to March 2014)

Eating speed			Quickly	Not quickly	X2^a^	df	effect size	p value^a^
Men			(n = 53,533)	(n = 131,991)				
	Smoking status	Current	24,171 (45.2)	62,985 (47.7)	223.568	2	0.035	< 0.001
		Former	11,154 (20.8)	23,691 (17.9)				
		Never	18,208 (34.0)	45,315 (34.3)				
	Alcohol intake	Everyday	21,403 (40.0)	57,443 (43.5)	195.729	2	0.033	< 0.001
		Sometimes	16,183 (30.2)	37,377 (28.3)				
		None	15,947 (29.8)	37,171 (28.2)				
	Daily physical activity	≥ 60 min	18,821 (35.2)	44,858 (34.0)	23.211	1	0.011	< 0.001
		< 60 min	34,712 (64.8)	87,133 (66.0)				
Women			(n = 22,162)	(n = 75,387)				
	Smoking status	Current	4698 (21.2)	14,410 (19.1)	215.489	2	0.047	< 0.001
		Former	1890 (8.5)	4702 (6.2)				
		Never	15,574 (70.3)	56,275 (74.7)				
	Alcohol intake	Everyday	3712 (16.7)	11,434 (15.2)	47.605	2	0.022	< 0.001
		Sometimes	6288 (28.4)	20,841 (27.6)				
		None	12,162 (54.9)	43,112 (57.2)				
	Daily physical activity	≥ 60 min	7313 (33.0)	23,236 (30.8)	37.69	1	0.02	< 0.001
		< 60 min	14,849 (67.0)	52,151 (69.2)				

df, degrees of freedom

Data are presented as n (%)

^a^Chi-squared test

The relationships of eating speed with an AST/ALT ratio of < 1 and ALT > 40 U/L according to sex are shown in Table 3. Eating quickly was significantly associated with a low AST/ALT ratio in both men (OR: 1.53, 95% CI: 1.50–1.56) and women (OR: 1.36, 95% CI: 1.31–1.41), as well as with an elevated ALT in both men (OR: 1.45, 95% CI: 1.41–1.49) and women (OR: 1.34, 95% CI: 1.25–1.43). Even after adjustment for age, γ-GTP, smoking status, alcohol intake, and physical activity, similar results were observed in both sexes. The strength of the associations of eating quickly with the AST/ALT ratio < 1 and ALT > 40 U/L was attenuated after further adjustment for BMI in model 3.

**Table 3:** Association of eating quickly with AST/ALT ratio < 1 or ALT > 40 (Japan, from April 2013 to March 2014)

		Not eating quickly group	Eating quickly group	Model 1		Model 2		Model 3	
				OR (95%CI)	p value	OR (95%CI)	p value	OR (95%CI)	p value
Men		(n = 131,991)	(n = 53,533)						
	AST/ALT ratio < 1, n (%)	58,290 (44.2)	29,307 (54.7)	1.53 (1.50–1.56)	< 0.001	1.44 (1.41–1.47)	< 0.001	1.08 (1.06–1.11)	< 0.001
	AST/ALT ratio ≥ 1, n (%)	73,701 (55.8)	24,226 (45.3)	1.00		1.00		1.00	
	ALT > 40, n (%)	17,113 (13.0)	9502 (17.7)	1.45 (1.41–1.49)	< 0.001	1.44 (1.40–1.48)	< 0.001	1.07 (1.04–1.11)	< 0.001
	ALT ≤40, n (%)	114,878 (87.0)	44,031 (82.3)	1.00		1.00		1.00	
Women		(n = 75,287)	(n = 22,162)						
	AST/ALT ratio < 1, n (%)	13,459 (17.9)	5040 (22.7)	1.36 (1.31–1.41)	< 0.001	1.37 (1.32–1.43)	< 0.001	1.11 (1.06–1.15)	< 0.001
	AST/ALT ratio ≥ 1, n (%)	61,928 (82.1)	17,122 (77.3)	1.00		1.00		1.00	
	ALT > 40, n (%)	3127 (4.1)	1213 (5.5)	1.34 (1.25–1.43)	< 0.001	1.42 (1.32–1.53)	< 0.001	1.09 (1.01–1.18)	< 0.001
	ALT ≤40, n (%)	72,260 (95.9)	20,949 (94.5)	1.00		1.00		1.00	

*BMI* body mass index, *AST* aspartate amino transferase, *ALT* alanine amino transferase

γ-GTP, γ-glutamyl transpeptidase; OR, odds ratio; CI, confidence interval

Model 1: No adjustment

Model 2: Adjusted for age, γ-GTP, smoking status, alcohol intake and physical activity

Moedl 3: Adjusted for age, γ-GTP, smoking status, alcohol intake, physical activity and BMI

The association between eating quickly and a low AST/ALT ratio according to the ALT level is presented in Table 4. In all models, a significant association between eating quickly and a low AST/ALT ratio was seen in men, regardless of ALT level, but it was seen only in women with ALT ≤40 U/L. On the other hand, in the adjusted models, a significantly increased ORs for an AST/ALT ratio < 1 was not observed in women with ALT > 40 U/L.

**Table 4:** Association of eating quickly with AST/ALT ratio < 1 among participants with ALT > 40 or not (Japan, from April 2013 to March 2014)

		Not eating quickly group	Eating quickly group	Model 1		Model 2		Model 3	
				OR (95%CI)	p value	OR (95%CI)	p value	OR (95%CI)	p value
Men									
ALT > 40		(*n* = 17,113)	(*n* = 9502)						
	AST/ALT ratio < 1, n (%)	15,338 (89.6)	8985 (94.6)	2.01 (1.81–2.23)	< 0.001	1.49 (1.33–1.66)	< 0.001	1.18 (1.05–1.33)	0.004
	AST/ALT ratio ≥ 1, n (%)	1775 (10.4)	517 (5.4)	1.00		1.00		1.00	
ALT ≤40		(*n* = 114,878)	(*n* = 44,031)						
	AST/ALT ratio < 1, n (%)	42,952 (37.4)	20,322 (46.2)	1.44 (1.40–1.47)	< 0.001	1.38 (1.35–1.41)	< 0.001	1.09 (1.06–1.12)	< 0.001
	AST/ALT ratio ≥ 1, n (%)	71,926 (62.6)	23,709 (53.8)	1.00		1.00		1.00	
Women									
ALT > 40		(*n* = 3127)	(*n* = 1213)						
	AST/ALT ratio < 1, n (%)	2802 (89.6)	1114 (91.8)	1.30 (1.03–1.65)	0.027	1.18 (0.92–1.51)	0.185	1.06 (0.83–1.36)	0.638
	AST/ALT ratio ≥ 1, n (%)	325 (10.4)	99 (8.2)	1.00		1.00		1.00	
ALT ≤40		(*n* = 72,260)	(*n* = 20,949)						
	AST/ALT ratio < 1, n (%)	10,657 (14.7)	3926 (18.7)	1.33 (1.28–1.39)	< 0.001	1.35 (1.30–1.41)	< 0.001	1.11 (1.07–1.16)	< 0.001
	AST/ALT ratio ≥ 1, n (%)	61,603 (85.3)	17,023 (81.3)	1.00		1.00		1.00	

*BMI* body mass index, *AST* aspartate amino transferase, *ALT* alanine amino transferase

γ-GTP, γ-glutamyl transpeptidase; OR, odds ratio; CI, confidence interval

Model 1: No adjustment

Model 2: Adjusted for age, γ-GTP, smoking status, alcohol intake, physical activity

Moedl 3: Adjusted for age, BMI, γ-GTP, smoking status, alcohol intake, physical activity

## Discussion

Eating quickly was associated with an elevated ALT and a low AST/ALT ratio in both men and women in the present study. The association persisted even after adjusting for age, γ-GTP, smoking status, alcohol intake, and physical activity. To the best of our knowledge, this was the first study to investigate the relationships of eating quickly with an elevated ALT and a low AST/ALT ratio. However, the findings of the present study need to be carefully considered.

ALT was reported to be positively associated with eating quickly, but the association disappeared after adjusting for BMI [8]; this implied that the association between eating quickly and ALT was dependent on BMI. On the other hand, the present study showed that the association between eating quickly and an elevated ALT remained even after adjusting for BMI. Two reasons need to be considered in an attempt to explain this difference in the results. One may be the larger number of participants in the present study, compared with that in the previous study. The other reason was the inclusion of energy intake in the previous study [8], but not in the present analysis. In this present study, eating quickly was also associated with a low AST/ALT ratio, which has been reported to be indicative of NASH [3, 15]. Therefore, modification of eating speed may decrease the risk for an elevated ALT and a low AST/ALT ratio and prevent NAFLD/NASH.

There are several mechanisms that may explain the associations of eating quickly with an elevated ALT and a low AST/ALT ratio. First, eating quickly might contribute to overweight/obesity, which can lead to an elevated ALT and a low AST/ALT ratio. Previous studies showed that eating quickly led to excess energy intake [7] and being overweight [16]. Moreover, ALT was shown to be significantly higher in overweight subjects than in non-obese subjects [17], and adults with an AST/ALT ratio < 1 had higher BMI, compared with the BMI of those with an AST/ALT ratio ≥ 1 [15]. These previous findings confirmed that the present study results were reasonable. Second, insulin resistance was reported to be associated with eating quickly and an elevated ALT and had a negative correlation with the AST/ALT ratio, based on the Homeostatic Model Assessment for Insulin Resistance (HOMA-IR) [15, 18, 19]. Therefore, eating quickly could lead to insulin resistance, which can contribute to an elevated ALT and a low AST/ALT ratio.

For men, the association between eating quickly and a low AST/ALT ratio was observed, regardless of the ALT level. Therefore, modification of the eating speed would be beneficial to prevent a low AST/ALT ratio, regardless of the ALT level. On the other hand, in women, the association between eating quickly and a low AST/ALT ratio was found in those with ALT ≤40 U/L, but not in those with ALT > 40 U/L. Therefore, in women, the benefit of eating speed modification in preventing a low AST/ALT ratio may be obtained in those with ALT ≤40 U/L. The reason for the absence of a significant association between eating quickly and a low AST/ALT ratio in women with ALT > 40 U/L could be the small number of participants with ALT > 40 U/L. Further studies will be needed to elucidate the biological mechanisms of this association.

This study had several limitations. First, eating speed was self-reported. Nevertheless, the use of a self-reported questionnaire to evaluate eating speed has been shown to be valid. The self-reported eating speed was well correlated with that reported by a friend [20] and with a decreased total number of chews [21]. Second, though the associations between an elevated ALT or a low AST/ALT ratio were statistically significant, further investigation is needed to see if it is significant in clinical or healthcare settings. Third, since this was a cross-sectional study, no causal relationship between eating quickly and liver enzymes can be established. Finally, the inclusion of study participants who were middle-aged Japanese men and women may limit the generalizability of our findings to other populations.

## Conclusions

In conclusion, eating quickly was significantly associated with an elevated ALT and a low AST/ALT ratio, regardless of sex. In addition, the association of eating quickly with a low AST/ALT ratio was seen even in individuals who had no ALT elevation. The present study suggested that modification of eating speed may contribute to reducing the risks for an elevated ALT and a low AST/ALT ratio and help prevent for NAFLD/NASH.

## Data Availability

The data used in the present study are available on reasonable request and only after approval by the Ethics Committee of the All Japan Labor Welfare Foundation.

## Abbreviations

ALT: Alanine aminotransferase
AST: Aspartate aminotransferase
AST/ ALT ratio: Aspartate aminotransferase to alanine aminotransferase ratio
OR: Odds ratio
CI: Confidence interval
NASH: Nonalcoholic steatohepatitis
NAFLD: Nonalcoholic fatty liver disease
BMI: Body mass index
γ-GTP: γ-glutamyl transpeptidase
HOMA-IR: Homeostatic Model Assessment for Insulin Resistance
